# Effect of 3% diquafosol sodium eye drops on the prediction of intraocular lens power in predisposition to dry eye patients scheduled for cataract surgery: a prospective, observational study

**DOI:** 10.3389/fmed.2025.1653439

**Published:** 2025-11-14

**Authors:** Na Su, Lexin Ge, Nianfeng Tang, Junjie Shan, Wen Fan, Songtao Yuan

**Affiliations:** 1 Department of Ophthalmology, The First Affiliated Hospital of Nanjing Medical University, Nanjing, Jiangsu, China; 2 Department of Ophthalmology, Xuzhou First People’s Hospital, Xuzhou, Jiangsu, China; 3 Department of Ophthalmology, The Affiliated Taizhou People’s Hospital of Nanjing Medical University, Taizhou, Jiangsu, China

**Keywords:** intraocular lens power calculation, cataract surgery, predisposition to dry eye, biological indexes, keratometry

## Abstract

**Purpose:**

To investigate the effect of 3% diquafosol sodium eye drops (DQS) on intraocular lens (IOL) power calculated by multiple common formulas before surgery in patients with predisposition to dry eye (p-DE) scheduled for cataract surgery.

**Methods:**

This prospective, observational study included patients scheduled for cataract surgery at the Ophthalmology Clinic of Jiangsu Provincial People’s Hospital between July 2022 and July 2023. A total of 50 eyes underwent repeated measurements to assess instrument stability, while 91 were divided into p-DE and control groups with mean tear break-up time (mBUT) <10 s and mBUT ≥10 s, respectively. Biological indexes were measured 5 min after DQS use in both p-DE and control groups.

**Results:**

Following DQS eye drops application, the p-DE group exhibited a higher number of eyes with changes in IOL power (calculated by SRK formula) and tear film stability (TFS) compared with the control group (*p* < 0.05) and the IOL power calculated by Hoffer Q formula also showed a statistical difference before and after DQS use (*p* < 0.05). After using DQS, the tear meniscus height (TMH), the first breakup time and the mBUT all increased in the p-DE group (*p* < 0.05), yet they were still lower than those in the control group. However, no significant differences were found in axial length, *K* value, corneal astigmatism axis, difference vector, anterior chamber depth, central corneal thickness, lens thickness, and white-to-white among all groups before and after DQS use (*p* > 0.05). Combined correlation analysis and logistic regression analysis revealed that changes in steep keratometry and TMH after treatment with DQS eye drops were the main factors affecting IOL power change. Additionally, mBUT before DQS use was identified as the primary factor affecting TFS change.

**Conclusion:**

Use of 3% DQS induces changes in intraocular lens power by affecting steep keratometry values, with such change being more significant in predisposition to dry eyes and warranting attention. When planning cataract surgery, it is recommended to prioritize the Barrett Universal II formula for IOL power calculation.

## 1 Introduction

Cataract phacoemulsification combined with intraocular lens (IOL) implantation is an effective method to restore vision in patients with cataracts. This procedure has evolved from a simple vision restoration surgery to precise refractive surgery. However, a refractive error of ±0.5 D is found postoperatively in 20–40% of patients with IOL (1). Reducing postoperative refractive errors is considered a key and challenging aspect of surgery. The discrepancy between the actual postoperative refractive error and the preoperative expected refractive error in cataract surgery may be attributed to axial length (AL) assessment (54%), anterior chamber depth (ACD) (38%), and keratometry (8%) (2). Although the iterative updates of the biometry devices have considerably reduced measurement errors, these errors become more significant in case of preoperative corneal irregularities or unstable tear film (1).

Dry eye disease (DED) incidence increases with age, with a prevalence between 5 and 50% (3). DED leads to decreased tear film stability, resulting in higher variability during preoperative biometry for cataract surgery. This may require multiple repeated measurements and even the use of artificial tears to stabilize the tear film for continued measurements (1). However, studies have reported that artificial tears may affect keratometry (K) measurements in DED, thereby impacting IOL power calculation. This measurement variability is most pronounced between baseline and 30 s and decreases over time. After 5 min of artificial tear use, accurate and repeatable keratometry measurements are obtained, improving optical visual quality, which should be considered during preoperative evaluation (1, 4, 5). Indeed, detecting DED in cataract cases preoperatively and treating DED with 0.09% cyclosporin and 0.05% cyclosporin A improves keratometry measurements and other biometric values, enhancing the accuracy of IOL power calculation (6–8).

Diquafosol ophthalmic solution (DQS) 3% is a P2Y2 receptor agonist that promotes tear fluid and mucin secretion without altering corneal thickness (9, 10). It is a new drug for DED that significantly improves tear break-up time (BUT) and higher-order aberrations. Compared with 0.3% sodium hyaluronate and cyclosporin A, DQS makes it easier to alleviate postoperative dry eye discomfort, especially for patients with foreign body sensation, reading difficulties, and issues with using video terminals, improving visual function (3, 11, 12). However, although the mechanism and effectiveness of DQS in DED are well established, no reports have examined the impact of DQS on IOL power calculation in patients with DED even predisposition to dry eye (p-DE) scheduled for cataract surgery. According to the TFOS DEWS II (Tear Film & Ocular Surface Society Dry Eye Workshop II), the presence of clinical signs (BUT shortening) without subjective symptoms is classified as a p-DE (13).

Human and animal pharmacokinetic studies found that DQS is rapidly degraded in the eye with a short residence time; in addition, the pH and osmotic pressure ratio of DQS are 7.2–7.4 and 1.0–1.1, respectively, which are close to those of tears under physiological conditions (14). Consequently, it is indicated that DQS is close to the physiological state, does not affect corneal thickness, and has short intraocular residence time. This suggests that DQS can be used to observe IOL power changes in different ocular surface states before cataract surgery.

We hypothesized administering DQS before biometry may improve ocular surface irregularities by stabilizing the tear film, which may lead to more stable and accurate keratometry measurements, thereby helping observe IOL power changes due to DED or p-DE. Thus, this study investigates the impact of DQS on biometric parameters and IOL power calculation in p-DE cases scheduled for cataract surgery, aiming to enhance the accuracy of IOL power calculation in p-DE.

## 2 Methods

A prospective, observational study was conducted at the Ophthalmology clinic of Jiangsu Provincial People’s Hospital. In this study, the recruitment period started on July 1, 2022, and ended on July 1, 2023. The study was approved by the Medical Ethics Committee of the First Affiliated Hospital of Nanjing Medical University (2022-SR-337). All methods implemented in this study were conducted in accordance with relevant guidelines and regulations. Participants were informed of all examinations involved and provided with signed informed consent forms. According to the TFOS DEWS II (Tear Film & Ocular Surface Society Dry Eye Workshop II) 2017, all eyes were divided into the p-DE group [mean BUT (mBUT) <10 s] and control group (mBUT ≥10 s) (3, 13, 15).

Inclusion criteria were: ① senile cataract scheduled for cataract surgery in our outpatient department; ② age over 40 years; ③ signed informed consent to cooperate with the examination. Exclusion criteria were: ① eyes with previous cataract surgery; ② any keratopathy; ③ use of any eye drops 24 h prior to examination; ④ corneal or conjunctival infection; ⑤ lacrimal apparatus or lacrimal duct disease; ⑥ systemic disease or eye disease affecting eye examination; ⑦ other types of cataracts, including congenital cataract, complicated cataract, etc. ⑧ a history of eye surgery or trauma.

**Figure 1:**
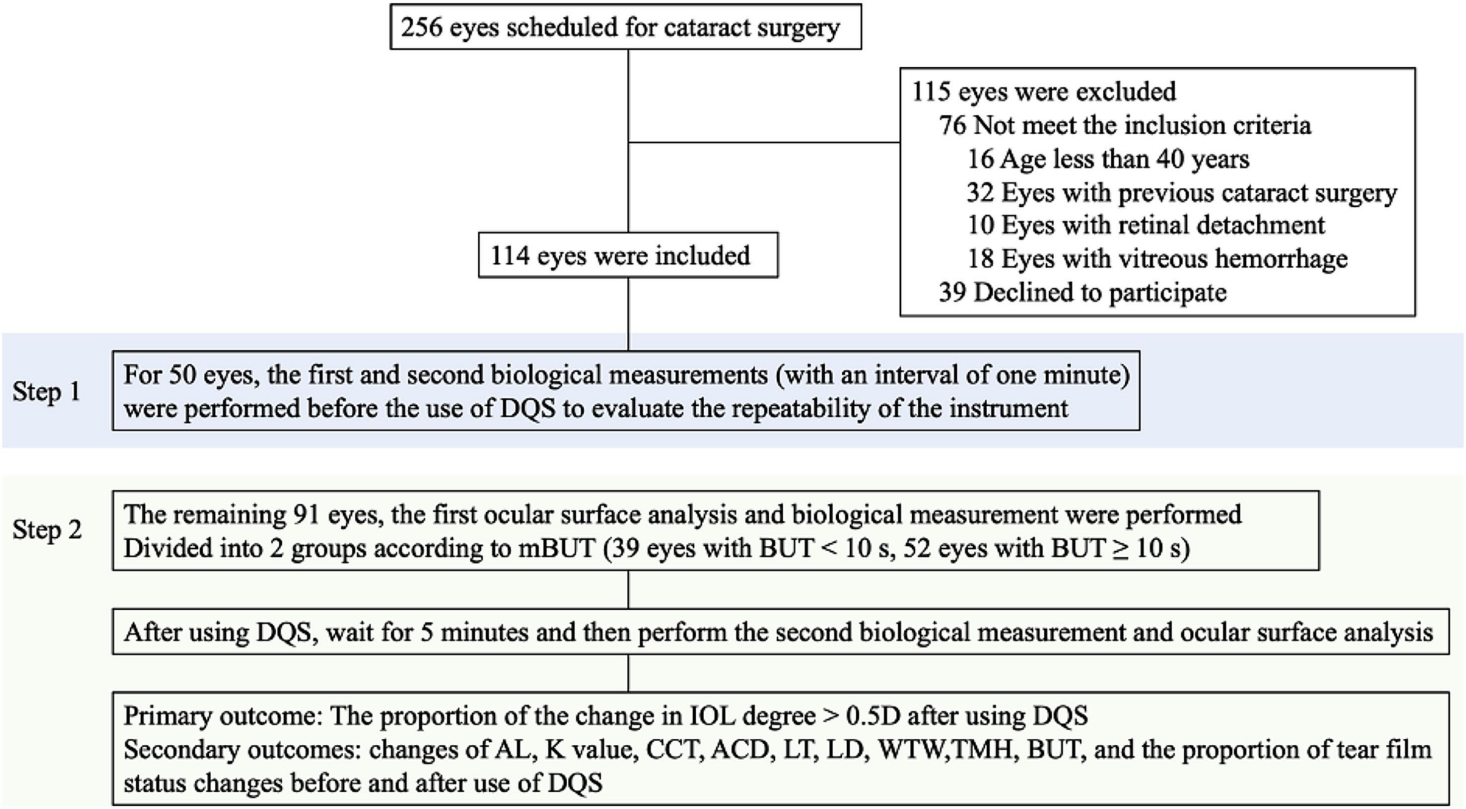
Study flowchart and procedures. DQS, diquafosol ophthalmic solution; AL, axial length; K, keratometry; ACD, anterior chamber depth; LT, lens thickness; LD, lens degree; CCT, central corneal thickness; WTW, white to white distance; TMH, tear meniscus height; mBUT, mean tear break-up time.

Medical history collection and slit-lamp fundus examinations were performed for 256 eyes scheduled for cataract surgery. Based on the above inclusion and exclusion criteria, 115 eyes were excluded, resulting in 141 eyes (77 patients) being included in the final analysis. Of these eyes, 50 underwent twice biological examination (IOL Master 700, Carl Zeiss Meditec AG, Jena, Germany) examinations at 1-min intervals to assess the stability and reliability of the instrument. Additionally, ocular surface analysis (OCULUS Keratograph 5M, Typ 77000, Germany) was performed for 91 eyes. Subsequently, all eyes were classified into the predisposition to dry eye (p-DE) group, characterized by a mean tear break-up time (mBUT) <10 s, and the control group with mBUT ≥10 s. Then the eyes were subjected to biological examination, with 1 drop of 3% DQS eye drop (Santen Pharmaceutical Co., Ltd., Noto Plant, China) placed in the conjunctival sac. Further eye surface analysis and biological measurement were performed after 5 min. Finally, the changes in the proportion of IOL power and diverse biological parameters were compared across the different groups (Figure 1). Both examinations were performed by the same doctor between 8:00 a.m. and 1:00 p.m., recording results according to the instrument’s built-in calculation method. The above tests were performed 3 times and an averaged value was recorded by the same ophthalmologist (NS).

The ocular surface analysis was used to acquire tear meniscus images and BUT in patients after blinking. The TMH directly below the central pupil was measured using a self-installed measurement tool. However, the head position of the patient was correct with both eyes staring straight ahead. After the central point was aligned with the pupil, the patient blinked twice. The patient was then asked to keep the eyes open until BUT measurement.

The reference IOL power in this study was calculated using the SRK regression formula (*P* = *A* − 2.5*L* − 0.9*K*), where *P* represents the intended IOL power, *A* is a constant (which depends on IOL type), *L* is the AL, and *K* is a keratometry index, which includes K1 (flat *K*) and K2 (steep *K*), representing different curvature values in two perpendicular directions on the cornea. In addition, we compared changes in IOL power using other common cataract IOL calculation formulas, including Hoffer Q and Barrett Universal II (Supplementary Table S4), via online calculators.^1^

The selection of IOL models was solely for standardized simulated preoperative assessment, used to uniformly analyze the impact of eye drops on calculated IOL power values, and does not represent the actual IOL models implanted in patients. In the initial analysis and the Barrett Universal II formula analysis, the ZCB00 aspheric IOLs was chosen, while the Alcon SN60WF/SA60WF lens was referenced in the Hoffer Q formula analysis. This specific pairing of models with formulas was implemented to better suit eyes of different ALs, thereby improving the clinical relevance and accuracy of the simulated IOL power calculations.

The primary outcome was the number of eyes with a change in IOL power prediction in p-DE patients following DQS use (IOL power change defined as a difference in IOL power of ≥0.5D between pre- and post-DQS measurements). Secondary outcomes were changes in axial length (AL), K1, K2, K2-K1, central corneal thickness (CCT), ACD, lens thickness (LT), white-to-white distance (WTW), tear meniscus height (TMH), first BUT (fBUT), mBUT, and eyes of tear film stability (TFS) change before and after DQS use (TFS change was defined as a switch of mBUT from <10 s to ≥10 s or from ≥10 s to <10 s). In addition to analyzing the corneal astigmatism *K*-values, we also referenced the vector analysis proposed by Alpins (16) to compare the comprehensive changes in corneal *K*-values, astigmatism axes (CAA) and difference vector (DV) before and after the use of DQS (The specific methods have been added in Supplementary material 7).

### 2.1 Sample size calculation

We used the pwr.p.test() function from the pwr package in R (RStudio, 2024.04.2 Build 764) to calculate the minimum sample size required for analyzing the proportional difference in IOL refractive power changes before and after DQS application. A total of 20 eyes were randomly selected in the preliminary study. The proportions of eyes with IOL power change ≥0.5D were 0.7 and 0.3 in the control and experimental groups, respectively. With the double-sided *z*-test used to test the rater difference between the two groups, the type l error was set to 0.05, and the sample ratio between the experimental and control groups was 1.0. Consequently, at least 21 eyes were required each in the control and p-DE groups to have a statistical power of 0.8 and reach a conclusion pointing to a rate difference between the two groups.

### 2.2 Statistical analysis

Exploratory data analysis and the Shapiro–Wilk test were performed to determine the normality of data distribution in p-DE and control groups. Continuous variables were expressed as mean ± standard deviation (SD) or median and interquartile range (IQR). Categorical variables were reported as count and percentage. Biological measurement and ocular surface analysis before and after eye drop application were compared by the paired samples t-test or non-parametric test (paired Wilcoxon signed-rank test). The independent samples t-test and non-parametric test (Mann–Whitney *U* test) were used to compare the p-DE and control groups. Count data in the p-DE and control groups were compared by the chi-square test. Spearman correlation analysis was used to determine the correlations between LD before and after eye drop instillation, the change in LD, and various parameters. Age and gender were used as covariates. Binary logistic regression was performed with biological measurement parameters as independent variables and IOL power change as the dependent variable to assess the relationship and determinants of IOL power change while accounting for age and gender. The level of significance was set as a 2-sided *p*-value below 0.05. All analyses were conducted with SPSS version 29.0 (SPSS Inc.).

## 3 Results

### 3.1 Baseline patient data

This study included a total of 141 eyes, with 50 undergoing repeated measurements to compare the instrument’s stability and reliability. The results showed that the biological instrument is stable with no changes in parameters and can be used for further analysis (Supplementary Table S1).

There were no significant differences in eye number, gender and age between the control and p-DE groups (*p* > 0.05) (Table 1).

**Table 1:** Baseline patient data.

General characteristics	Eye/sex	Control group (*n*)/mean ± SD	p-DE group (*n*)/mean ± SD	*p*
Eye^b^	Right eye	24	20	0.628
	Left eye	28	19	
Gender^b^	Male	23	17	0.951
	Female	29	22	
Age^a^		64.79 ± 12.55	65.44 ± 12.88	0.810

p-DE, predisposition to dry eye.

a Paired *t*-test.

b Chi-square test.

### 3.2 IOL power changes after DQS use

**Figure 2:**
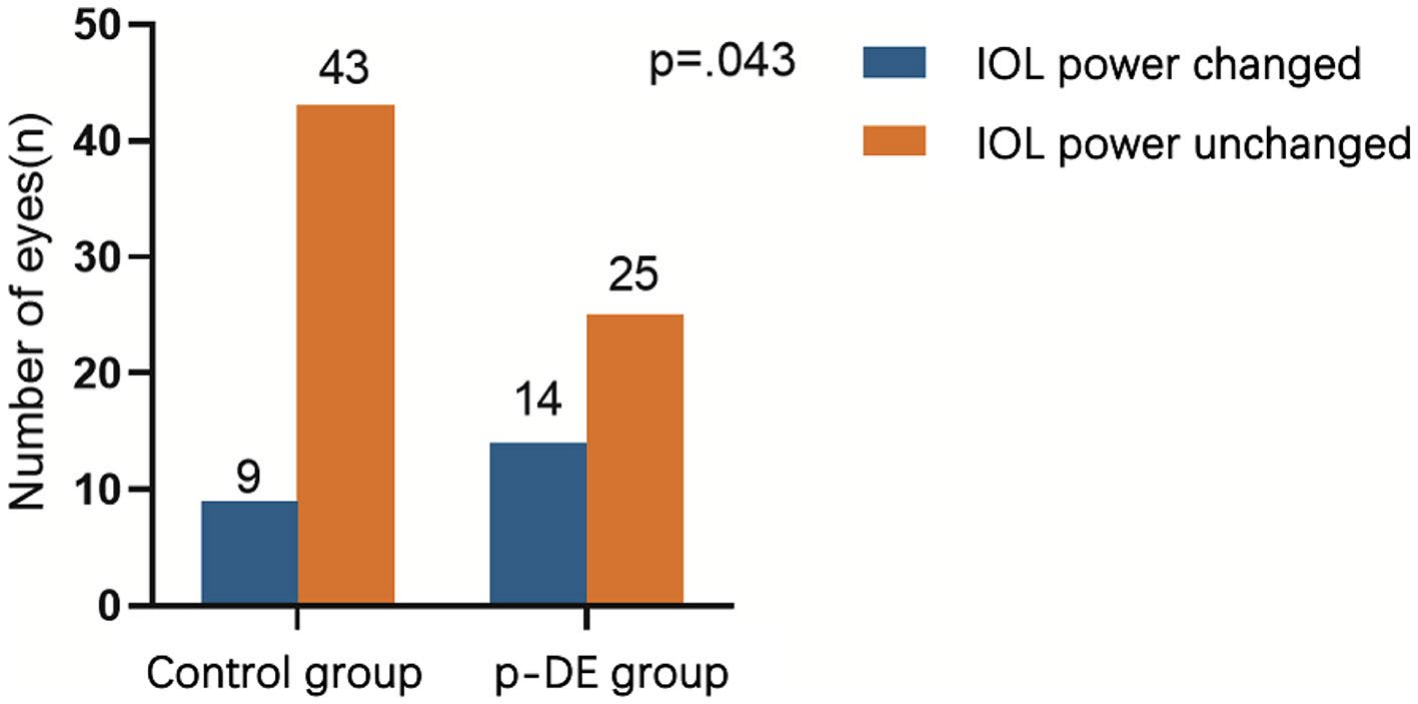
IOL power changes after DQS use. The number of eyes with IOL power change was significantly higher in the p-DE group than in the control group.

Before and after DQS use, median IOL power in the p-DE and control groups was 19.5 diopters. In p-DE group, IOL power increased by 0.5 diopter (D) in 7 eyes, decreased by 0.5 D in 6 and decreased by 1 D in 1. In the control group, IOL power increased by 0.5 D in 3 eyes, decreased by 0.5 D in 5, and increased by 1 D in 1. After DQS use, the number of eyes with IOL power change was significantly higher in the p-DE group than in the control group [14 (35.9%) vs. 9 (17.3%), *p* < 0.05] (Figure 2).

In p-DE eyes, 4 male eyes (23.5%) and 10 female eyes (45.5%) exhibited changes, while 13 male eyes (76.5%) and 12 female eyes (54.5%) showed no change (*p* > 0.05) (see Supplementary Table S3). In addition, we compared IOL power changes via other common cataract IOL calculation formulas (Hoffer Q and Barrett Universal II; Table 2) and found no significant intergroup difference between the p-DE and control groups (*p* > 0.05). However, within the p-DE group, IOL power calculated by the Hoffer Q formula increased significantly before and after DQS use (*p* < 0.05) (see Supplementary Table S5).

**Table 2:** Changes in IOL power calculated by different formulas after DQS use.

Formulas	IOL power changed	Control/*n* (%)	p-DE/*n* (%)	$\chi^{2}$	*p*
SRK^a^	Changed	9 (17.3)	14 (35.9)	4.078	**0.043** ^*^
	Unchanged	43 (82.7)	25 (64.1)		
Hoffer Q^a^	Changed	13 (25)	12 (30.8)	0.372	0.542
	Unchanged	39 (75)	27 (69.2)		
Barrett II^a^	Changed	18 (34.6)	9 (23.1)	1.422	0.233
	Unchanged	34 (65.4)	30 (76.9)		

Change in IOL power: refractive power change ≥ 0.5D before and after drop instillation.

a Chi-square test. Bold or * indicates *P* < 0.05.

The number of eyes with tear film stability change was also higher in the p-DE group compared with the control group [13 (33.3%) vs. 8 (15.4%), *p* < 0.05] (Supplementary Table S2).

### 3.3 Changes in biological parameters after treatment in p-DE group

After using DQS eye drops, TMH, fBUT, and mBUT were all increased in p-DE group (*p* < 0.05), while the remaining parameters showed no significant differences (*p* > 0.05) (Table 3).

**Table 3:** Changes in biological parameters after treatment in p-DE patients.

Parameter	Pretreatment with DQS mean ± SD/Median (IQR)	Post-treatment with DQS mean ± SD/Median (IQR)	*t*/*Z*	*p*
AL^b^	23.76 (1.34)	23.72 (1.34)	159	0.788
K1^a^	43.78 ± 1.4	43.70 ± 1.42	1.287	0.144
K2^a^	44.77 ± 1.77	44.75 ± 1.76	−0.043	0.513
K2-K1^b^	0.85 (0.71)	0.97 (0.87)	415.5	0.514
CAA^b^	91 (93)	94 (60.5)	3,434	0.375
ACD^a^	3.04 ± 0.43	3.07 ± 0.49	−0.85	0.439
LT^a^	4.57 ± 0.45	4.57 ± 0.45	−0.843	0.567
CCT^a^	543.77 ± 34.85	544.33 ± 33.34	−1.355	0.506
WTW^a^	11.60 ± 0.50	11.62 ± 0.52	−1.015	0.572
TMH^b^	0.18 (0.12)	0.20 (0.12)	424	0.01^*^
fBUT^b^	4.01 (2.48)	6.12 (3.64)	487.5	0.005^*^
mBUT^b^	6.15 (4.24)	7.70 (8.22)	489.5	0.004^*^

^*^p < 0.05. p-DE, predisposition to dry eye. DQS, diquafosol ophthalmic solution; AL, axial length; K, keratometry; K1, flat keratometry; K2, steep keratometry; CAA, corneal astigmatism axis; ACD, anterior chamber depth; LT, lens thickness; CCT, central corneal thickness; WTW, white to white distance; TMH, tear meniscus height; fBUT, first tear break-up time; mBUT, mean tear break-up time.

a Paired *t*-test.

b Paired Wilcoxon signed-rank test.

### 3.4 Changes in biological parameters after treatment in control group

After using DQS eye drops, TMH was significantly higher than the pretreatment value (0.20 vs. 0.24, *p* < 0.01) in the control group, while the remaining parameters showed no significant differences (*p* > 0.05) (Table 4).

**Table 4:** Changes in biological parameters after treatment in control group.

Parameter	Pretreatment with DQS mean ± SD/median (IQR)	Post-treatment with DQS mean ± SD/median (IQR)	*t*/*Z*	*p*
AL^b^	23.81 (2.54)	23.82 (2.56)	272	0.628
K1^a^	43.78 ± 1.11	43.77 ± 1.08	0.263	0.794
K2^a^	44.64 ± 1.23	44.66 ± 1.18	0.54	0.591
K2-K1^b^	0.81 (0.63)	0.82 (0.52)	654	0.873
CAA^b^	85 (78)	91 (85.25)	1223.5	0.535
ACD^a^	3.08 ± 0.41	3.08 ± 0.41	0.493	0.624
LT^a^	4.41 ± 0.45	4.42 ± 0.45	0.927	0.358
CCT^a^	530.42 ± 25.77	531.33 ± 26.69	1.194	0.238
WTW^b^	11.8 (0.6)	11.8 (0.47)	400	0.887
TMH^b^	0.20 (0.14)	0.24 (0.16)	266.5	0.003^*^
fBUT^b^	13.29 (13.24)	11.88 (12.57)	472	0.788
mBUT^b^	18.43 (8.27)	16.43 (9.42)	362	0.079

^*^p < 0.05. DQS, diquafosol ophthalmic solution; AL, axial length; K, keratometry; K1, flat keratometry; K2, steep keratometry; CAA, corneal astigmatism axis; ACD, anterior chamber depth; LT, lens thickness; CCT, central corneal thickness; WTW, white to white distance; TMH, tear meniscus height; fBUT, first tear break-up time; mBUT, mean tear break-up time.

a Paired *t*-test.

b Paired Wilcoxon signed-rank test.

### 3.5 Comparison of parameters in different groups

Both before and after using DQS, the fBUT (16.82 vs. 4.4) and the mBUT (21.02 vs. 8.03) were significantly higher in the control group compared with the p-DE group. No statistically significant differences were found in CCA and DV difference between the p-DE and control groups (*p* > 0.05), and there was no correlation between DV and IOL power change (*p* > 0.05) (Table 5).

**Table 5:** Comparison of IOL master parameters in all eyes.

	Pretreatment with DQS				Post-treatment with DQS			
Parameter	Control group mean ± SD/median (IQR)	p-DE group mean ± SD/median (IQR)	*t*/*U*	*p*	Control group mean ± SD/median (IQR)	p-DE group mean ± SD/median (IQR)	*t*/*U*	*p*
AL^b^	23.89 (2.39)	23.64 (1.73)	827	0.244	23.89 (2.35)	23.64 (1.65)	831	0.258
K1^a^	43.64 ± 1.07	43.86 ± 1.33	−0.802	0.425	43.62 ± 1.03	43.81 ± 1.34	−0.687	0.494
K2^a^	44.41 ± 1.00	44.86 ± 1.69	0.058	0.116	44.43 ± 0.94	44.86 ± 1.67	−1.563	0.122
K2-K1^b^	0.77 (0.56)	0.86 (0.73)	1063.5	0.438	0.80 (0.54)	0.89 (0.64)	1075.5	0.382
CAA^b^	85 (78)	91 (93)	2716.5	0.438	91 (85.25)	94 (60.5)	2569.5	0.667
DV^b^	0.17 (0.29)	0.26 (0.23)	2,844	0.069	—	—	—	—
ACD^a^	3.09 ± 0.43	3.04 ± 0.41	0.548	0.585	3.10 ± 0.43	3.06 ± 0.45	0.367	0.715
LT^a^	4.43 ± 0.38	4.51 ± 0.50	−0.84	0.403	4.45 ± 0.38	4.51 ± 0.50	−0.616	0.54
CCT^a^	527.97 ± 21.85	536.47 ± 34.91	−0.149	0.882	537.00 ± 22.06	536.84 ± 34.40	0.027	0.979
WTW^b^	11.80 (0.40)	11.60 (0.60)	757.5	0.082	11.60 (0.60)	11.60 (0.50)	724.5	0.044
LD^b^	19.25 (7.63)	19.50 (6.50)	−0.147	0.883	19.50 (7.13)	19.50 (6.50)	−0.118	0.456
TMH^b^	0.20 (0.14)	0.18 (0.13)	919.5	0.684	0.22 (0.16)	0.23 (0.15)	1034.5	0.59
fBUT^b^	16.82 (9.90)	4.40 (3.48)	0	<0.001^*^	15.97 (13.99)	6.50 (5.64)	429.5	<0.001^*^
mBUT^b^	21.02 (7.19)	8.03 (6.51)	77	<0.001^*^	18.00 (10.41)	10.71 (9.41)	508	<0.001^*^

^*^p < 0.05. p-DE, predisposition to dry eye; DQS, diquafosol ophthalmic solution; AL, axial length; K, keratometry; K1, flat keratometry; K2, steep keratometry; CAA, corneal astigmatism axis; DV, difference vector (see Supplementary material 7 for details); ACD, anterior chamber depth; LT, lens thickness; CCT, central corneal thickness; WTW, white to white distance; LD, lens diopter; TMH, tear meniscus height; fBUT, first tear break-up time; mBUT, mean tear break-up time.

a Paired *t*-test.

b Mann–Whitney *U* test.

Spearman correlation analysis indicated that LD after DQS use was significantly negatively correlated with AL before DQS (*r* = −0.909, *p* < 0.001), K2 before DQS (*r* = −0.266, *p* = 0.011), AL after DQS (*r* = −0.910, *p* < 0.001), K2 after DQS (*r* = −0.256, *p* = 0.014), ACD before DQS (*r* = −0.551, *p* < 0.001), and ACD after DQS (*r* = −0.540, *p* < 0.001), and significantly positively correlated with TMH before DQS (*r* = 0.254, *p* = 0.015). The differences in TMH, fBUT, and mBUT before and after DQS use were significantly negatively correlated with their respective baseline values before DQS (*r* = −0.356, −0.469, −0.477, respectively, all *p* < 0.001), while the differences in the remaining parameters showed no correlation with their baseline values before DQS use (*p* > 0.05). The LD difference was significantly negatively correlated with the K1 difference (*r* = −0.446, *p* < 0.001), the K2 difference (*r* = −0.332, *p* = 0.001), and the AL difference (*r* = −0.269, *p* = 0.010) (See Supplementary Table S6 for details).

### 3.6 Factors affecting IOL power changes

Age and gender were used as covariates of adjustment parameters, and a binary stepwise logistic regression analysis was carried out. The results showed that while controlling the effects of age and gender, the *K* value and TMH after using DQS were the main factors affecting the change in intraocular lens power (Table 6).

**Table 6:** Logistic regression analysis of factors affecting IOL power and TFS changes.

IOL power change	*B*	OR (95% CI)	*p*
Sex (M)	−1.948	0.143 (0.037, 0.554)	0.005^*^
K2 (post-treatment)	0.682	1.978 (1.284, 3.048)	0.002^*^
TMH (post-treatment)	5.178	177.269 (1.318, 23837.563)	0.038^*^
Constant	−32.564	0.000	0.001
Tear film stability change			
mBUT (pretreatment)	−0.089	0.915 (0.846, 0.989)	0.025^*^
Constant	−0.086	0.917	0.862

^*^p < 0.05. K2, steep keratometry; TMH, tear meniscus height; mBUT, mean tear break-up time. Sex, was only a covariate to control its potential impact on the outcome variable, thereby enabling a more accurate assessment of the independent effect of biological parameters on IOL power. It was not discussed as an influencing factor.

Meanwhile, mBUT before using DQS eye drops was the main factor affecting tear film stability change (Table 6).

## 4 Discussion

In this study, biological measurements were performed again at 5 min after DQS application, referring to the conclusion by Röggla et al. (1). They found that in cataract patients, after using artificial tears of different viscosities, the variability of keratometric measurements and astigmatism fluctuations both decreased to clinically acceptable ranges at 5 min, with a stable tear film ensuring measurement accuracy. This study revealed a high repeatability for parameters in both baseline measurements before DQS use, consistent with studies by Röggla and Yue Peng reporting high repeatability for AL, Km, K1, K2, ACD, LT, CCT, and WTW (1, 17).

Compared with healthy individuals, preoperative corneal measurements in cataract patients with p-DE have higher inaccuracy. Liu and Pflugfelder (18) found that the chronic dry state and immune activation in DED result in decreased central and peripheral corneal thickness, while artificial tears or cycloplegia may increase thickness (17), improve higher-order aberrations on the anterior corneal surface, help temporarily restore corneal surface regularity and tear film stability (19), and enhance corneal optical quality and vision. In contrast to the above studies, this research observed no changes in CCT or WTW after DQS use versus pretreatment values, in line with reports by Momeni-Moghaddam et al. (20) who found no statistical differences in WTW, AL, and CCT following cycloplegia. These discrepant results may be related to the inclusion of normal or mild dry eye patients in the study.

Other studies have also assessed the impacts of artificial tears, cycloplegia, and intraocular pressure-lowering medications on biometric parameters, demonstrating that eye drops do not affect AL or keratometry measurements (1, 17), and preoperative biometry correlates with subjective refraction at 6 weeks postoperatively (21). This study found that in patients with p-DE using DQS eye drops, there were no significant changes in AL and *K* values, corroborating the above findings. However, our study found no significant difference in CAA before and after DQS use in either the p-DE group or the control group, which is consistent with the study by Mrukwa Kominek et al. (22), who performed corneal topography on patients with ocular surface disease aged >50 years. In the 20–50 years group and the normal control group, corneal astigmatism value first increased and then decreased with the extension of blink time, while no intergroup difference was observed in astigmatism axis, which is consistent with the results of our study. Although we did not follow up the patients to determine the actually implanted IOLs or postoperative visual acuity, the DV values were consistent with those previously reported by Xu et al. (23) at 3 months postoperatively (approximately 0.3), and also consistent with Alió et al. (24), who found no significant difference in DV during the 6-month follow-up after IOL implantation.

This study also demonstrated that DQS does not alter LT and ACD. Previous reports suggested that LT may be linked to age and disease. In healthy individuals over 40 years old, LT tends to decrease with age (20). The thicker LT in cataract eyes compared with normal eyes may not be caused by lens opacity but rather by progressive lens growth due to aging (25).

Accurate assessment of ACD is crucial, as measurement errors can damage the corneal endothelium and lead to postoperative refractive errors. Previous reports have linked ACD to ethnicity, medications, ocular accommodation, and other ocular biometric parameters. Lam et al. (26) demonstrated that ACD is significantly shorter in Hispanic patients compared with non-Hispanic counterparts. Eye accommodation increases LT, causing the lens to move forward, while cycloplegia might result in unchanged or thinner LT and increased ACD by reducing lens curvature and shifting the geometric center backward (17, 20). This difference in LT is considered to be age-related.

Besides, factors associated with ACD include AL, LT, and WTW, with LT being the primary factor affecting ACD, followed by AL (27). ACD is negatively correlated with LT and positively correlated with AL and WTW (28). These changes should be considered when determining IOL power to prevent refractive errors after cataract surgery.

We found that the proportion of eyes with changes in IOL power by SRK forula was significantly higher in the p-DE group than in the control group, whereas no such changes were observed with the Hoffer Q and Barrett Universal II formulas. However, the IOL power calculated by the Hoffer Q formula in the p-DE group increased significantly after DQS administration. The Hoffer Q formula assigns a significantly higher weight to K-value than the SRK formula and does not incorporate multi-parameter dynamic correction like the Barrett II formula; we attribute these observed differences to the varying sensitivities of the three formulas to *K*-value fluctuations. The results of this study are consistent with previous studies: Jiang et al. (29) found that eyes with unstable tear film had greater variability in anterior segment measurement parameters, especially when calculating IOL power using the SRK formula, while the Barrett Universal II formula yielded more stable results in such eyes; Röggla et al. (1) also reported that high-viscosity eye drops could cause 13.2 and 34.4% changes in IOL power in normal individuals and dry eye patients, respectively. Although we did not follow up the postoperative refractive power in patients with IOL power changes, scholars proposed that active treatment (such as rebamipide ophthalmic suspension and lifitegrast 5% eye drops) used in DED treatment not only improves superficial corneal punctate keratopathy, BUT, and higher-order aberrations but also enhances the accuracy of IOL power prediction in patients scheduled for cataract surgery (30, 31), which will be the direction of our future in-depth research.

This study found that after using DQS eye drops, the p-DE group showed significantly increased TMH, fBUT, and mBUT, while the control group only showed an increase in TMH. This is in line with previous reports indicating that DQS eye drops are effective in dry eye treatment in humans and mouse models, significantly improving tear production, BUT, higher-order aberrations, and subjective symptoms (32–34), with more pronounced improvements in individuals over 60 years old (35). According to our results, the worse the baseline BUT, the more significant the improvement effect of the drug. The potential mechanism involves DQS binding to specific receptors to induce mucin secretion from goblet cells, thereby facilitating rapid corneal epithelial repair and restoring ocular surface integrity under the protection of mucins.

Furthermore, five minutes after using DQS eye drops, there was a significant increase in tear sialic acid levels without protein dilution similar to saline, addressing the issue of tear dilution while ensuring treatment efficacy (36). DQS and similar drugs such as rebamipide clear solution are effective options for improving dry eye and post-cataract surgery dry eye by enhancing BUT, tear volume, and lipid layer thickness (37, 38).

After the use of DQS, K2 and TMH were identified as the main factors influencing the changes in IOL power, with age and gender taken into account. Meanwhile, the mBUT before the use of DQS was a primary factor affecting p-DE. Previous findings have also demonstrated the impacts of anterior segment depth (ASD, ACD + LT) and cycloplegia on IOL power, revealing that individuals with deeper ASD tend to have predicted lens powers leaning towards hyperopia (39). Formulas for IOL power calculation, apart from the Olsen formula, showed no significant changes after cycloplegia, with significant negative correlations determined with AL and ACD. The increase in ACD is thought to be related to the optical biometry equipment applied (40).

Previous studies have shown that factors such as a shorter BUT in the DED group lead to a decrease in the reproducibility of corneal measurements, thereby affecting the calculation of IOL power (4, 41). Correlation analysis showed LD after DQS use was significantly correlated with AL, K2, and ACD (before and after DQS use) and TMH before DQS use, which is closely related to the calculation formula of LD. Although there were no significant differences in AL, ACD, and K2 before and after DQS use, minor changes in these values may be reflected in the comprehensive LD results. Of course, although LD was closely correlated with steep *K*, there was no difference in LD before and after DQS use, while the number of patients with IOL changes in the p-DE group was significantly higher than that in the control group. Such differences might be related to the fact that the subjects in the study were in the predisposition to dry eye rather than having an obvious and confirmed diagnosis of dry eye disease, or it is also uncertain whether this change has real clinical significance remains uncertain, and large-sample prospective studies are still needed for verification in the future.

To the best of our knowledge, this study is the first to find that preoperative use of DQS in cataract patients with p-DE may affect ocular surface conditions, thereby influencing IOL power selection and postoperative refraction. Although there were no statistically significant differences in corneal *K*-values, CAA or DV before and after the use of DQS, and there was no correlation between DV and IOL power change, changes in IOL power are closely associated with the steep *K*-value.

### 4.1 Limitations

First, patients were divided into p-DE and control groups based on mBUT, without assessing the effects of other artificial tears of different concentrations or saline solutions on IOL power, which may have similar impacts on the evaluated variables and should be explored in further research. In addition, we only focused on the potential effect of DQS on IOL power in predisposition to dry eye cases with cataracts, the selection of these IOL models was solely for standardized simulated preoperative assessment, therefore, we did not perform follow-up to assess the consistency between predicted and postoperative IOL power in patients. Owing to the lack of data on actual IOL implantation and postoperative refraction, it was not possible to evaluate the link between the eye drop use and the final refractive outcome. Prospective randomized controlled studies should be conducted in the future, focusing on enrolling participants across multiple AL ranges to validate the results of multiple commonly used IOL calculation formulas both before and after eye drop administration, verifying the dose–response relationship between eye drop administration and changes in ocular parameters, and comparing the impact (including IOL astigmatism and CAA variation) of eye drop use on the actually implanted IOLs. Additionally, it is necessary to evaluate corneal punctate epithelial staining, higher-order aberrations, symptom questionnaire scores, and cost-effectiveness comparisons of different eye drops. This is aimed at enhancing the accuracy of lens selection for cataract patients with dry eye and providing improved guidance for visual treatment.

In conclusion, the instability of IOL power after DQS use in p-DE patients may be related to tear film instability and changes in corneal curvature induced by the eye drops. Although this study did not observe the actual implanted IOL power in patients, nor analyze the difference between the formula-recommended IOL power and the actually used IOL power, or the postoperative refractive error, it still suggests that preoperative medication in p-DE cataract patients may significantly influence IOL selection, even though this study cannot yet determine whether this influence is positive or negative. Close attention should be paid to p-DE patients in clinical practice. When planning cataract surgery, it is recommended to prioritize the Barrett Universal II formula for IOL power calculation, so as to reduce the impact of parameter fluctuations on IOL power calculation results.

## Data Availability

The original contributions presented in the study are included in the article/Supplementary material, further inquiries can be directed to the corresponding authors.

## Footnotes

^1^https://hofferqst.com and https://calc.apacrs.org/barrett_universal2105/
