# Supplementary material for: Effect of 3% diquafosol sodium eye drops on the prediction of intraocular lens power in predisposition to dry eye patients scheduled for cataract surgery: a prospective, observational study
Source: Front Med (Lausanne). 2025 Nov 14;12:1653439. doi: 10.3389/fmed.2025.1653439 (PMC12661514; doi:10.3389/fmed.2025.1653439)
Supplement: Supplementary file 1 [file Table_1.doc]

Effect of 3% diquafosol sodium eye drops on the prediction of intraocular lens power in predisposition to dry eye patients scheduled for cataract surgery: A prospective, observational study

Na Su^1¶^, Lexin Ge^2¶^, Nianfeng Tang^1^, Junjie Shan^3&*^, Wen Fan^1&*^, Songtao Yuan^1&*^

1. Repeatability and reliability testing of IOL Master instrument.

Supplementary Table S1 IOL Master instrument repeatability and reliability

| Parameter | The first examination  Mean ± SD/  median (IQR) | The second examination mean ± SD/  median (IQR) | t/ Z | *P* | ICC | *P* |
| --- | --- | --- | --- | --- | --- | --- |
| Eye（R） | 50 (23) | | - | - | - | - |
| Gender(M) | 50 (17) | | - | - | - | - |
| age | 64.92±12.17 | | - | - | - | - |
| AL$ | 23.80(3.04) | 23.79(3.04) | 126.5 | 0.486 | 1.000 | <0.001 |
| K1# | 43.88±1.17 | 43.87±1.17 | 0.429 | 0.67 | 0.991 | <0.001 |
| K2# | 44.68±1.26 | 44.68±1.27 | -0.097 | 0.923 | 0.996 | <0.001 |
| K2-K1$ | 0.72(0.51) | 0.76(0.58) | 339.5 | 0.653 | 0.937 | <0.001 |
| LD$ | 19.50(9.13) | 19.25(9.25) | 8 | 0.257 | 1.000 | <0.001 |
| ACD# | 3.10±0.42 | 3.10±0.42 | 1.218 | 0.229 | 1.000 | <0.001 |
| LT# | 4.40±0.46 | 4.40±0.46 | -1.429 | 0.159 | 1.000 | <0.001 |
| CCT# | 534.96±32.03 | 535.28±31.64 | -0.675 | 0.503 | 0.997 | <0.001 |
| WTW$ | 11.60(0.63) | 11.60(0.63) | 167 | 0.403 | 0.291 | <0.001 |

R: right. M: male. ICC: intra-class Correlation Coefficient. AL: axial length. K: keratometry. K1: flat keratometry. K2: steep keratometry. LD: lens diopter. ACD: anterior chamber depth. LT: lens thickness. CCT: central corneal thickness. WTW: white to white distance. #: paired t test. $: paired Wilcoxon signed-rank test.

2. IOL diopter and tear film stability changes before and after DQS use

Supplementary Table S2 Changes of IOL power and tear film stability after DQS use

| Parameter | Control group  n (%) | p-DE group  n (%) | 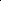 | *P* |
| --- | --- | --- | --- | --- |
| IOL power changed& | 9(17.3) | 14(35.9) | 4.078 | 0.043* |
| IOL power unchanged& | 43(82.7) | 25(64.1) |
| TFS changed& | 8(15.4) | 13(33.3) | 4.044 | 0.044* |
| TFS unchanged& | 44(84.6) | 26(66.7) |

IOL: intraocular lens. DQS: diquafosol ophthalmic solution. p-DE: predisposition to dry eye. TFS: tear film stability. &: Chi-square test.

3. Gender differences in intraocular lens power in the p-DE group

Supplementary Table S3. Comparison of changes in intraocular lens power between male and female in the p-DE group

| Eyes |  | Male (%) | Female (%) | 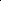 | *P* |
| --- | --- | --- | --- | --- | --- |
| p-DE group | IOL power changed & | 4 (23.5) | 10 (45.5) | 2.003 | 0.157 |
|  | IOL power unchanged & | 13 (76.5) | 12 (54.5) |  |  |

IOL: intraocular lens. p-DE: predisposition to dry eye. &: Chi-square test.

4. The Effect of DQS Eye Drops on a Variety of Commonly Used IOL Power Calculation Formulas

Supplementary Table S4 Selection of Commonly Used Formulas for IOL Power Calculation

| Formulas | Range of applicable ALs; Advantages & Disadvantages; Inclusion of anterior segment parameters |
| --- | --- |
| SRK/T | - Applicable to patients with regular ALs (22–26 mm) and demonstrates good predictability for long ALs. - Advantages: Widely used in clinical practice, easy to operate, and accounts for the nonlinear relationship between AL and IOL position. - Limitations: May have relatively large errors in patients with short ALs or high myopia. - Included parameters: AL and K values. |
| Barrett II | - Optimized based on multi-center big data, it is suitable for the full range of ALs, and particularly achieves higher accuracy than the Holladay 2, SRK/T, Hoffer Q, and Holladay 1 formulas in patients with long ALs (AL > 26 mm) [1-3]. - Advantages: Based on ray tracing technology, it accounts for changes in the principal plane of the IOL and offers high predictive accuracy. - Limitations: Accurate measurement of parameters such as ACD and LT is required. - Included parameters: AL, K values, ACD, LT and White to White. |
| Hoffer Q | - Suitable for patients with short ALs (< 22 mm) or shallow ACD [3], and is based on the principle of optical coherence. - Advantages: It has a strong ability to correct ACD and is applicable to cases with significant variations in corneal curvature and AL. - Limitations: May have relatively large errors in patients with long AL. - Included parameters: AL, K values and ACD. |

IOL: intraocular lens. AL: axial length. K: keratometry. ACD: anterior chamber depth. LT: lens thickness.

1. Changes in IOL power calculated by various formulas before and after application of DQS eye drops

Supplementary Table S5 Changes in IOL power calculated by various formulas before and after application of DQS eye drops

| IOL Power (D) |  | SRK | Hoffer Q | Barrett II |
| --- | --- | --- | --- | --- |
| p-DE (n=52) | DQS-pre | 16.30±8.66 | 17.21±8.78 | 17.54±8.23 |
|  | DQS-post | 16.26±8.65 | 17.31±8.77 | 17.54±8.32 |
|  | P# | 0.399 | **0.020** | 0.896 |
| Control (n=39) | DQS-pre | 16.04±6.63 | 16.88±6.79 | 16.90±6.65 |
|  | DQS-post | 16.07±6.62 | 16.85±6.87 | 16.97±6.68 |
|  | P# | 0.488 | 0.624 | 0.201 |
| All (n=91) | DQS-pre | 17.30±6.69 | 17.09±8.05 | 17.30±7.64 |
|  | DQS-post | 17.30±6.70 | 17.14±8.07 | 17.32±7.71 |
|  | P# | 0.824 | 0.151 | 0.640 |

DQS: diquafosol ophthalmic solution. IOL: intraocular lens. p-DE: predisposition to dry eye. #: paired t test.

1. Correlations between parameter changes and baseline values.

Supplementary Table S6 Spearman Correlation Analysis Between Parameter Changes and Pre-DQS Parameter Values

| Correlation analysis parameter pairs | Correlation coefficient (r) | P value |
| --- | --- | --- |
| AL1 vs AL2 - AL1 | -0.014 | 0.895 |
| K1_1 vs K1_2 - K1_1 | -0.112 | 0.289 |
| K2_1 vs K2_2 – K2_1 | -0.147 | 0.166 |
| ACD1 vs ACD2 - ACD1 | -0.114 | 0.281 |
| LT1 vs LT2 - LT1 | -0.014 | 0.896 |
| CCT1 vs CCT2 - CCT1 | -0.09 | 0.395 |
| WTW1 vs WTW2 - WTW1 | -0.137 | 0.194 |
| LD1 vs LD2 – LD1 | -0.121 | 0.253 |
| TMH1 vs TMH2 – TMH 1 | -0.356 | **<0.001** |
| fBUT1 vs fBUT2 - fBUT1 | -0.469 | **<0.001** |
| mBUT1 vs mBUT2 - mBUT1 | -0.477 | **<0.001** |
| LD2 – LD1 vs AL2 - AL1 | -0.269 | **0.010** |
| LD2 – LD1 vs K1_2 - K1_1 | -0.446 | **<0.001** |
| LD2 – LD1 vs K2_2 – K2_1 | -0.332 | **0.001** |

DQS: diquafosol ophthalmic solution. AL: axial length. K: keratometry. K1: flat keratometry. K2: steep keratometry. LD: lens diopter. ACD: anterior chamber depth. LT: lens thickness. CCT: central corneal thickness. WTW: white to white distance. TMH, tear meniscus height; fBUT, first tear break-up time; mBUT, mean tear break-up time.

7. Alpins vector analysis

The Alpins vector analysis method [4] is often used to determine the effectiveness of astigmatism correction in laser refractive surgery. In this study, adding vector analysis allows us to upgrade from "simply observing the numerical change in astigmatism" to "integrating the magnitude and direction of astigmatism to accurately assess the impact of corneal curvature on IOL power".

**Specific method for calculating the** **difference vector (DV) of** **astigmatism**

DV represents the difference between the "astigmatism vector after eye drop application" and the "astigmatism vector before eye drop application". The calculation involves 3 steps: ① Convert the astigmatism parameters into vector components (x, y) in the Cartesian coordinate system. Astigmatism is essentially a two-dimensional vector. It is necessary to convert "polar coordinates (C, θ)" to "Cartesian coordinates (x, y)" using trigonometric functions to eliminate the nonlinear influence of the axis angle. The formulas are as follows:

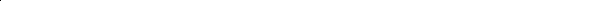


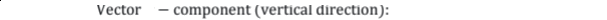


Example: A patient's astigmatism was
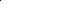
,
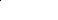
before the eye drops and
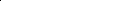
after.

According to the formulas above, we obtain:

Preoprative vector
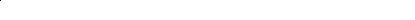


Preoprative vector
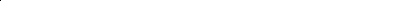


Postoprative vector
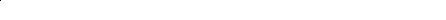


Postoprative vector
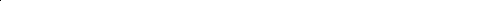


② Calculate the difference in vector components before and after eye drop application (Δx, Δy), i.e., the post-instillation vector components minus the pre-instillation vector components, reflecting the change of the vector in the x and y directions:


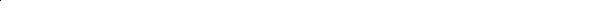


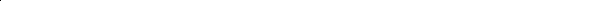


According to the formulas above, we obtain:


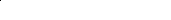


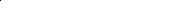


③ Calculate the magnitude of the DV of astigmatism. DV is the hypotenuse of the right triangle formed by Δx and Δy, representing:


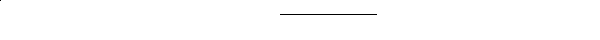


According to the formula above, we calculated that:


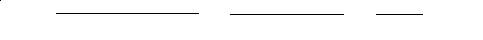


Reference

[1] Melles RB, Holladay JT, Chang WJ. Accuracy of Intraocular Lens Calculation Formulas. Ophthalmology. 2018 Feb;125(2):169-178. doi: 10.1016/j.ophtha.2017.08.027. Epub 2017 Sep 23. PMID: 28951074.

[2] Wang Q, Jiang W, Lin T, Zhu Y, Chen C, Lin H, Chen W. Accuracy of intraocular lens power calculation formulas in long eyes: a systematic review and meta-analysis. Clin Exp Ophthalmol. 2018 Sep;46(7):738-749. doi: 10.1111/ceo.13184. Epub 2018 Mar 24. PMID: 29498180.

[3] Hoffer KJ, Savini G. IOL Power Calculation in Short and Long Eyes. Asia Pac J Ophthalmol (Phila). 2017 Jul-Aug;6(4):330-331. doi: 10.22608/APO.2017338. PMID: 28780778.

[4] Alpins N. Astigmatism analysis by the Alpins method. J Cataract Refract Surg. 2001 Jan;27(1):31-49. doi: 10.1016/s0886-3350(00)00798-7. PMID: 11165856.
